# Supplementary material for: Complexome profiling of the Chlamydomonas psb28 mutant reveals TEF5 as an early PSII assembly factor
Source: Plant Cell. 2025 Mar 17;37(6):koaf055. doi: 10.1093/plcell/koaf055 (PMC12164590; doi:10.1093/plcell/koaf055)
Supplement: koaf055_Supplementary_Data [file koaf055_supplementary_data.zip › psb28_interactive.zip!/plots/intensity/qualityPlots/peptides/psbL_Cre-1.g2716995.t1.p1.html]

### Cre-1.g2716995.t1.p1

Trivial name: psbL  
SeqCoverage [median]: 36.80  
Mass: 4.43 kDa  
  
 

| Sequence | MissedCleavage | Mass [Da] | wtIntensity | muIntensity | Score |
| --- | --- | --- | --- | --- | --- |
| ARPNPNK | 1 | 795.44 | 846072.05 | 162498.10 | 167.98 |
| ARPNPNKQVVELNR | 2 | 1633.90 | 14248.88 | 2284.39 | 102.73 |
| QVVELNR | 0 | 856.48 | 222539.05 | 39278.29 | 193.98 |
|  |  |  | 1082860.0 | 204060.8 |  |
